# Inclusion of people of color in psychedelic-assisted psychotherapy: a review of the literature

**DOI:** 10.1186/s12888-018-1824-6

**Published:** 2018-07-31

**Authors:** Timothy I. Michaels, Jennifer Purdon, Alexis Collins, Monnica T. Williams

**Affiliations:** 1 Department of Psychological Sciences, University of Connecticut, Bousfield Psychology Building, 406 Babbidge Road, Unit 1020, Storrs, CT 06269 USA; 2 Department of Psychiatry, University of Connecticut Health Center, 263 Farmington Avenue, Farmington, CT 06030 USA

**Keywords:** Psychedelic-assisted psychotherapy, Ethnic differences, Minority recruitment, People of color

## Abstract

**Background:**

Despite renewed interest in studying the safety and efficacy of psychedelic-assisted psychotherapy for the treatment of psychological disorders, the enrollment of racially diverse participants and the unique presentation of psychopathology in this population has not been a focus of this potentially ground-breaking area of research. In 1993, the United States National Institutes of Health issued a mandate that funded research must include participants of color and proposals must include methods for achieving diverse samples.

**Methods:**

A methodological search of psychedelic studies from 1993 to 2017 was conducted to evaluate ethnoracial differences in inclusion and effective methods of recruiting peopple of color.

**Results:**

Of the 18 studies that met full criteria (*n* = 282 participants), 82.3% of the participants were non-Hispanic White, 2.5% were African-American, 2.1% were of Latino origin, 1.8% were of Asian origin, 4.6% were of indigenous origin, 4.6% were of mixed race, 1.8% identified their race as “other,” and the ethnicity of 8.2% of participants was unknown. There were no significant differences in recruitment methodologies between those studies that had higher (> 20%) rates of inclusion.

**Conclusions:**

As minorities are greatly underrepresented in psychedelic medicine studies, reported treatment outcomes may not generalize to all ethnic and cultural groups. Inclusion of minorities in futures studies and improved recruitment strategies are necessary to better understand the efficacy of psychedelic-assisted psychotherapy in people of color and provide all with equal opportunities for involvement in this potentially promising treatment paradigm.

## Background

### The promise of psychedelic medicine

The use of altered or non-ordinary states of consciousness for medicinal purposes is neither novel nor modern, but rather dates back thousands of years to the spiritual practices of indigenous communities across the world. For indigenous peoples, psychedelic use is considered a both a sacred and healing act, that requires the guidance of a highly trained spiritual leader (shaman), and entails psychoactive rituals that bring humans closer to the spiritual world, in an effort to treat both physical and spiritual ills [1]. While the full history of indigenous healing practices has been covered elsewhere [2], understanding its roots within the historical origins of psychedelic-assisted psychotherapy serves as an important reference point, given that modern psychedelic medicine has struggled to include marginalized communities, especially people of color, in this movement, and is only now beginning to acknowledge the importance of their inclusion [3–5].

Western medicine’s exploration of psychedelics for treatment purposes can be divided into two distinct periods, with the first occurring between 1950 and 1985 (herein referred to as the “first wave,”), when synthetic psychedelic compounds were just being discovered, and the second (herein referred to as the “second wave”) beginning around the late 1990s and continuing to this day [6]. This periodic distinction has been observed by others The “rediscovery” of psychedelics as medicine by Western science first occurred during a period in which biomedical therapeutic interventions in psychiatry were limited, as psychopharmacology had not yet become mainstream practice [7]. Newly synthesized psychedelics were not considered controlled substances, and therefore their clinical and research use was relatively unrestrained. Given that psychoanalysis was a mainstay of treatment, initial research on psychedelic medicine examined whether psychedelic drugs could facilitate the process of psychotherapy, thereby accelerating the treatment process of psychological disorders [8]. Yet ultimately, the combination of widespread use of these substances, serious ethical violations (i.e., administration to physically-restrained subjects, sexual abuse between therapists and clients), major methodological flaws, and concerns over safety led to defunding of research and ultimately the scheduling of psychedelics as controlled substances [8, 9]. While many researchers and subjects continued to espouse the therapeutic benefits of psychedelic-assisted therapy for the treatment of depression, addiction, and other disorders, this area of investigation lay dormant for several decades.

Several important factors contributed to the resurgence of psychedelic medicine in the late 1990s following a several decade-long hiatus. Instead of launching into therapeutic investigations, early research during the second wave consisted of pre-clinical animal and basic science studies that were carefully conducted to establish the pharmacological properties and safety of these substances [2, 8]. With the creation of the Multidisciplinary Association of Psychedelic Studies (MAPS), a non-profit pharmaceutical organization, by Rick Doblin in 1986, researchers were no longer reliant upon government funding and could pursue FDA drug development [10]. The medicinal potential for scheduled substances was also no longer novel, given that research into the healing properties of both cannabis [11, 12] and ketamine [13] had been underway since 1975. Coupled with the fact that many psychiatric illnesses still lacked effective pharmacological treatment, these factors paved the way for the second wave of psychedelic research.

Recent renewed interest in psychedelic-assisted psychotherapy has benefited from avoiding the mistakes of the past by focusing explicitly on ethical, methodological and clinical safety issues. It has also taken a careful and gradual approach to re-introducing this controversial area of study, working closely with regulatory bodies and federal agencies [10]. Indeed, initial results from second-wave (2000-present) psychedelic research has demonstrated the efficacy of psilocybin [14–16] for the treatment of depression, addiction, and OCD, LSD [14, 17–19] in the treatment of depression, anxiety, and substance use, ayahuasca [20, 21] for the treatment of depression and addiction, and MDMA [22, 23] in reducing trauma symptoms. Yet these promising findings have often been limited to small, predominantly White samples, limiting the generalizability of findings and excluding people of color from potential therapeutic benefits. Beyond a broad lack of representation of people of color in psychedelic-assisted psychotherapy studies, the conceptualization of psychopathologies rarely include important cultural considerations such as the importance of including race-based trauma when recruiting participants of color for MDMA-assisted psychotherapy for PTSD [24]. In the United States, this lack of inclusion goes directly against federally mandated efforts to report and recruit diverse samples in clinical trials.

The following study aims to provide a comprehensive review of inclusion and recruitment across ethnic/racial groups in current (1993-present) psychedelic-assisted psychotherapy studies, in an effort to characterize the scope and importance of this issue while identifying areas for growth. To provide the necessary context around these issues, we first review the prevalence and presentation of psychopathology in people of color and the importance of cultural considerations in the design and implementation of clinical trial research. Upon reporting findings on the rates of inclusion and recruitment strategies in psychedelic studies, we conclude by summarizing current efforts to acknowledge diversity issues in the field and provide considerations for future directions.

### Prevalence of psychological disorders across people of color

Population-level prevalence rates of psychopathology are often underestimated in people of color [25], and variability between studies can be driven by differences across age groups, in socio-economic status, and in study sampling methodologies [26]. Yet converging evidence suggests that in the US, people of color experience psychological distress at a rate equal to and sometimes higher than non-Hispanic Whites. For instance, the lifetime prevalence of PTSD in Black Americans is 8.03%, which is higher than the prevalence rate in Hispanic/Latino Americans (5.59%), Asian Americans (1.84%), or Non-Hispanic Whites (6.45%) [27].

While the lifetime prevalence of substance dependence (drug and alcohol) in Hispanic Americans is comparable to the prevalence in Black Americans, the rate of Generalized Anxiety Disorder (GAD) of 5.89% is higher than any other ethnic group, with the exception of non-Hispanic Whites [27]. Notably, there are some disorders for which Hispanics are at a relatively lower risk, including dysthymia, oppositional-defiant disorder, and attention deficit hyperactivity disorder [28].

Although Asian Americans generally report lower lifetime prevalence rates for most psychological disorders [29], a recent study reported that they experience OCD at a similar rate to Black Americans [30]. For Asian Americans, the lifetime prevalence of experiencing a severe depressive episode is also comparable to that for Black Americans [31].

Despite adequate data on the lifetime prevalence of psychopathology in Black Americans, there remain discrepancies in accurate diagnosis that further reinforce barriers to treatment. Although several studies report similar lifetime prevalence rates of OCD between non-Hispanic Blacks and other ethnic groups [28, 30, 32], the diagnosis is often missed during structural clinical interviews, reducing enrollment in treatment trials [33–35]. For Black Americans the issue of appropriate diagnosis and recruitment extends to anxiety disorders as well [36] and despite similar lifetime prevalence rates of a major depressive episode in Black Americans [37] compared to Non-Hispanic White Americans [28], there are large disparities in treatment attrition rates [38].

Overall, it is evident that people of color experience significant rates of psychological distress comparable to non-Hispanic whites, and furthermore experience significant barriers to treatment that likely increase the risk for long-term negative outcomes. It is therefore critical that novel treatment paradigms include minorities in all stages of research thereby improving our understanding of efficacy in people of color and improves changes of treatment effectiveness in the community.

### Symptom presentation in people of color

The unique clinical presentation of mental illnesses in people of color is often overlooked in the hegemonic, White U.S. mental health care system. This may be due, in part, to long-standing social and economic barriers to psychological treatments for minorities [39, 40] and a shameful history of the medical establishment subjecting minorities to significant harm as a direct result of medical research participation [41]. For instance, one study reported that the underutilization of mental health services by undocumented Latinos exposed to intimate partner violence (IPV) can partially be explained by common healthcare barriers among immigrants, including language, insurance, economic barriers, and documentation status (Perez and Fortuna 2005). The impact of these factors cannot be understated when attempting to reconcile the great need for targeted mental health treatment for people of color and low rates of engagement with the mental healthcare system. While a full discussion of these factors is beyond the scope of this review, and has been covered extensively elsewhere [39–41], it provides a framework for considering barriers to recruitment in psychedelic science research and for the importance of incorporating culturally specific conceptualizations of clinical presentation.

Both such external constructionist factors and intrinsic factors, such as differences in the manifestation and clinical presentation of psychological symptoms in people of color, likely contribute to difficulties in generalizing clinical trial design and recruitment. Race-based trauma and PTSD symptoms provides an especially relevant example of the unique factors related to the pathogenesis of psychopathology in people of color and well as diverse clinical presentations that are often not considered by clinicians and researchers. Microaggressions are forms of aversive racism that, whether intentional or not, communicate hostile or negative racial biases and insults to a target person or group [42, 43]. These racial slights may be perceived as harmless, but they are considered a form of everyday discrimination and racism that induce emotional and traumatic stress responses in those afflicted. In a study of Latinos, traumatic stress responses were the mechanisms by which microaggressions contributed to symptoms of depression and anxiety [44]. Discrimination is also associated with increased anxiety, affective, and substance abuse disorders among African Americans, Hispanic Americans, and Asian Americans [45]. Exposure to race and ethnic based discrimination is related to increased lifetime prevalence of generalized anxiety disorder in African Americans [46]. Yet despite the common contribution of racism to PTSD symptomatology across people of color, it would be a mistake to assume equivalency in clinical presentation, as recent studies have highlighted important differences in how individuals of varying ethnicities response to traumatic experiences [47]. For these reasons, it is important for psychedelic research to address the relevance of ethnic and race-based trauma symptoms in relation to mental illnesses across different ethnoracial groups. The invalidation and avoidance of racial-cultural issues by clinicians has had detrimental consequences on relationships between mental health practitioners and their patients or clients of color [48].

The role of ethnic identity is another important factor that often ignored in mental health care settings. Low ethnic identity in African Americans has been linked to poor self-esteem, problems with adjustment, poor achievement, delinquency, eating disorders, and substance abuse [49, 50]. Although African Americans report a significantly stronger ethnic identity than European Americans [49], there are still many cultural and systemic barriers that limit access to mental health care and lead to the underutilization of these services in the African American community. Given the above, it is not only crucial that mental health providers examine the relationship between race and ethnic based discrimination and psychopathology but also incorporate an understanding of and explicit discussion of ethnic identity in conceptualization and treatment.

### Mandate for inclusion

In order to ensure that ethnic and racial minorities are adequately represented in research studies in the United States, Congress passed the *NIH Revitalization Act of 1993,* which mandates that minorities and women be included in representative numbers for all studies conducted or supported by the NIH [51]. The Act states that not only must recruitment be conducted in manner that is fair to persons from ethnic/racial minority groups, and they must be included in studies unless there is clear scientific evidence ruling out important clinical or public health importance for their inclusion [52, 53]. By 1995, the NIH refused to fund any project that did not adhere to such policies and required grant proposals to include strategies by which to achieve diversity in their samples. Following the 1993 act, diversity in NIH clinical research has increased on an absolute basis, from approximately 1 million minority participants in 1995 to almost 15 million in 2016. Yet on a relative basis, participation rates for people of color have been relatively stagnant, representing 36.7% of all enrollment in 1995 and 37.2% of all participants in 2016 [51, 54].

While these guidelines do not apply to research funded outside the NIH, and many psychedelic studies conducted in the USA are privately funded, the NIH mandate reflects the importance of this issue and should serve as a benchmark for non-government funded research within the US. To our knowledge, there are no similar mandates from government funding institutions in countries outside the United States. Yet given the importance of inclusion in clinical research, the prevalence of psychological disorders in other cultures, and mental health inequities that exist in all industrialized nations, we believe that the NIH mandate should serve as an international standard.

### Purpose of present investigation

The authors of this investigation sought to determine the rates of inclusion for people of color in psychedelic-assisted psychotherapy studies by conducting a review of such literature from 1993 to 2017. Our objective was to determine if the existing data on psychedelic treatments was sufficient to generalize to all people of color. Furthermore, given differences in the manifestation and clinical presentation of psychological symptoms in people of color, we were also interested in whether rates of inclusion reflected differences in recruitment methodology or in how pathology was conceptualized in the relevant research. Although the studies reviewed herein were conducted in many different countries, each with their own important histories related to historical disenfranchisement, oppression, and people of color representation, we believe the importance of ethnoracial diversity in psychological research writ-large and their inclusion in psychedelic science specifically transcends national boundaries. In providing recommendations going forward, we note the need for considering country-level differences in conducting research that will likely impact issues of inclusion.

## Methods

### Definition of psychedelics

Within the field of psychedelic science, there remains much debate regarding how to define what constitutes a “psychedelic” drug; differing definitions have important implications for the plants and compounds under clinical investigation. One common approach in defining psychedelic compounds is based on these substances’ neurobiological mechanism of action. Using this approach, psychedelics are generally categorized into two broad chemical classes: indolamines, such as psilocybin, DMT and LSD, which primarily act on monamine neurotransmitters such as serotonin (5-HT), and phenylalkylamines, such as MDMA and mescaline, that derive their name from their action on calcium channel blockage [55, 56].

A second approach to classification relies on the unique behavioral effects of these drugs. Behavioral Pattern Monitor (BPM) studies in rats have shown that psychedelics exhibit a distinguishable profile of behavioral effects analogous to the enhanced sensitivity and reactivity to environmental stimuli that occurs in humans [55]. For instance, results from a recent study on psychopathological, neuropsychological and personality differences between regular ayahuasca users and controls showed that individuals using ayahuasca regularly scored significantly higher on self-report measures of self-transcendence, and demonstrated increased cognitive enhancement when matched with controls [57].

Yet others [10] have argued that limiting the term psychedelics to the above definitions excludes certain substances, such as cannabis and ketamine, that subjectively induce “hallucinogenic” effects and are believed to have medicinal and clinical benefits [11, 13]. For the purpose of the present investigation, the term psychedelic is limited to compounds that primarily exert their effects through 5-HT agonist. These compounds are not only similar from a neurobiological perspective but also are also most consistently utilized in psychedelic-assisted psychotherapy, whereas other substances, such as cannabis and ketamine, not only differ substantially in their psychopharmacology, but can exert clinical benefits without a psychotherapy component.

### Selection of studies for inclusion

We performed a thorough and extensive search for studies that utilized a psychedelic substance either alone or in conjunction with psychotherapy in the treatment of a psychological disorder. For the purpose of the present study, psychological disorder was defined as any condition included in Axis 1 of the Diagnostic and Statistical Manual of Mental Disorders IV Text Revised (DSM-IV-TR) or the International Statistical Classification of Diseases and Related Health Problems 9th edition (ICD-9) equivalent. When applicable for more recent studies, the equivalent classification was determined based on DSM-5 or ICD-10 diagnostic codes. Given the early stages of research on psychedelic medicine, we included trials with varying methodology including qualitative, exploratory, preliminary, pilot, randomized, and placebo-controlled studies. Advanced searches were conducted using the PsychINFO, PubMed, and Scopus database engines. In addition to search terms specific to each substance, we included the following key terms within the title, keyword, and abstract of peer-reviewed published studies; “random,” “randomized,” “randomized control,” “controlled,” “controlled trial,” “qualitative,” “quantitative,” “pilot,” “psychedelic,” “psychedelic-assisted,” “psychedelic medicine.” We also searched the reference list of review articles on psychedelic medicine to find other studies that may meet inclusion criteria and obtained the relevant articles using the above-mentioned databases.

Studies conducted before 1993 were excluded from our review. This decision was made in part due to the *NIH Revitalization Act of 1993* in the United States and to exclude studies from the “first wave” of psychedelic research which lacked the methodological rigor and did not meet the standard of current the ethical clinical practices guidelines [8]. Given the global nature of the psychedelic medicine movement, we included research from any country in which studies were published in peer-reviewed journals and available in English.

### Missing data

The reporting of study participants’ ethnicity varies considerably [58], and this is especially true outside the United States. For studies that met the above inclusion criteria but did not disclose ethnoracial information in the publication (*n* = 10, 58.8%), we contacted the corresponding authors directly via email or phone call. When applicable, email correspondences were translated into the native language of the study’s country of origin. Several attempts were made to reach the corresponding author before reaching out to another author of the study (typically the first or second author of each respective study). If the missing data were provided, this information was included in the data analysis. In two instances, researchers provided data on participant ethnicity but indicated that this information was not obtained through self-report but rather by having research staff estimate participants’ ethnicity. Given the potential inaccuracies and prejudices of this approach [59], such data are listed as “unknown” and were excluded from data analysis. Substantial research suggests that estimates of ethnicity are often inaccurate for ethnic minorities, especially when compared to self-reported data.

### Compilation of research

Data collected from the selected studies were included in one master spreadsheet. Periodically the data were updated as study staff obtained previously missing information from study authors. For each study, we collected the following information: psychedelic substance, country and location (city and state when possible) where the study was conducted, study methodology (qualitative, quantitative, observational), randomization (open-label, single-blind, double-blind, placebo-controlled), number of participants, and participants’ ethnicity (total number and percent of total). Furthermore, we collected information on recruitment methods to determine whether such factors may have influenced minority participation.

As ethnic and racial identities differ substantially by country, minority status varies as well. Given that minority status is unique to each country’s historical, political and social contexts, we believe it is important to consider minority inclusion in psychedelic studies relative to the broader countrywide representation of such groups. Therefore, in addition to study specific data, we collected national demographic information for any country included in the final analysis. This demographic information was sourced from census data collected by the statistic or economics department of each country’s national government. An effort was made to include data that corresponded approximately to the time period in which each study was conducted.

### Data analysis

We conducted descriptive statistics to arrive at the percentage of inclusion by ethnoracial group for each study as well as summary statistics across all studies and by geography. Raw data were double entered by study staff to protect against possible errors and all variables of interest were dummy coded for statistical analysis purposes. Two-tailed Pearson and point biserial correlations were used to determine is there was a correlation with recruitment method variables and minority inclusion.

## Results

### Minority inclusion

A total of 18 studies met full criteria and were selected for inclusion in the present analysis. Seven studies (38.9%) were conducted in the United States, three studies (16.7%) in Brazil, two studies each (11.1%) from New Zealand, Switzerland, and the United Kingdom and one study (5.6%) each from Canada and Spain (Table 1). With respect to the primary psychedelic substances investigated, the results were as follows; psilocybin (eight studies, 44.4%), ayahuasca (four studies, 22.2%), MDMA (three studies, 16.7%), ibogaine (two studies, 11.1%) and LSD (one study, 5.6%). Studies for two of the five psychedelics were exclusively conducted within a single country; all ayahusca studies were conducted in Brazil and both ibogaine studies were from New Zealand. The sole LSD study was conducted in Switzerland. Seven studies (38.9%) utilized ayahuasca or psilocybin for the treatment of depression, five studies (27.8%) administered ayahuasca, psilocybin or ibogaine for the treatment of substance use disorders, four (22.2%) provided LSD or psilocybin for the treatment of an anxiety disorder, three (16.7%) administered MDMA-assisted psychotherapy for the treatment of post-traumatic stress disorder, and one study (5.6%) used psilocybin in the treatment of obsessive-compulsive disorder.

**Table 1:** Ethnoracial makeup of psychedelic medicine studies

Study	Psychedelic	Country	*N*	White	Black	Latino	Asian	Indigenous	Mixed	Other	Unknown
Osorio et al., 2015	Ayahuasca	Brazil	6	0(0.0%)	0(0.0%)	0(0.0%)	0(0.0%)	0(0.0%)	0(0.0%)	0(0.0%)	6(100.0%)
Palhano-Fontes et al., 2017	Ayahuasca	Brazil	29	17 (58.6%)	1 (3.4%)	0(0.0%)	0(0.0%)	0(0.0%)	11 (37.9%)	0(0.0%)	0(0.0%)
Sanches et al., 2016	Ayahuasca	Brazil	17	0(0.0%)	0(0.0%)	0(0.0%)	0(0.0%)	0(0.0%)	0(0.0%)	0(0.0%)	17 (100.0%)
Thomas et al., 2013	Ayahuasca	Canada	12	0(0.0%)	0(0.0%)	0(0.0%)	0(0.0%)	12 (100.0%)	0(0.0%)	0(0.0%)	0(0.0%)
Brown & Alper, 2017	Ibogaine	Mexico	30	27 (90.0%)	0(0.0%)	1 (3.3%)	0(0.0%)	0(0.0%)	0(0.0%)	2 (6.7%)	0(0.0%)
Noller, Frampton & Yazar-Klosinski, 2016	Ibogaine	New Zealand	14	14 (100.0%)	0(0.0%)	0(0.0%)	0(0.0%)	0(0.0%)	0(0.0%)	0(0.0%)	0(0.0%)
Bouso et al., 2008	MDMA	Spain	6	6(100.0%)	0(0.0%)	0(0.0%)	0(0.0%)	0(0.0%)	0(0.0%)	0(0.0%)	0(0.0%)
Gasser et al., 2014	LSD	Switzerland	11	11 (100.0%)	0(0.0%)	0(0.0%)	0(0.0%)	0(0.0%)	0(0.0%)	0(0.0%)	0(0.0%)
Oehen et al., 2013	MDMA	Switzerland	14	12 (85.7%)	0(0.0%)	0(0.0%)	1 (7.1%)	0(0.0%)	1 (7.1%)	0(0.0%)	0(0.0%)
Carhart-Harris et al., 2016	Psilocybin	UK	12	9 (75.0%)	2 (16.7%)	1 (8.3%)	0(0.0%)	0(0.0%)	0(0.0%)	0(0.0%)	0(0.0%)
Carhart-Harris et al., 2018	Psilocybin	UK	8	6 (75.0%)	1 (12.5%)	0(0.0%)	1 (12.5%)	0(0.0%)	0(0.0%)	0(0.0%)	0(0.0%)
Mithoefer et al., 2010	MDMA	USA	20	20 (100.0%)	0(0.0%)	0(0.0%)	0(0.0%)	0(0.0%)	0(0.0%)	0(0.0%)	0(0.0%)
Bogenschutz et al., 2015	Psilocybin	USA	10	3 (30.0%)	1 (10.0%)	4 (40.0%)	0(0.0%)	1 (10.0%)	0(0.0%)	0(0.0%)	0(0.0%)
Griffiths et al., 2016	Psilocybin	USA	51	48 (94.1%)	2 (3.9%)	0(0.0%)	1 (2.0%)	0(0.0%)	0(0.0%)	0(0.0%)	0(0.0%)
Grob et al., 2011	Psilocybin	USA	12	11 (91.7%)	0(0.0%)	0(0.0%)	1 (8.3%)	0(0.0%)	0(0.0%)	0(0.0%)	0(0.0%)
Johnson et al., 2014	Psilocybin	USA	15	14 (93.3%)	0(0.0%)	0(0.0%)	1 (6.7%)	0(0.0%)	0(0.0%)	0(0.0%)	0(0.0%)
Moreno et al., 2006	Psilocybin	USA	9	8 (88.9%)	0(0.0%)	0(0.0%)	0(0.0%)	0(0.0%)	1 (11.1%)	0(0.0%)	0(0.0%)
Ross et al., 2016	Psilocybin	USA	29	26 (89.7%)	0(0.0%)	0(0.0%)	0(0.0%)	0(0.0%)	0(0.0%)	3 (10.3%)	0(0.0%)
*N* = 18		Total	282.0	232 (82.3%)	7 (2.5%)	6 (2.1%)	5 (1.8%)	13 (4.6%)	13 (4.6%)	5 (1.8%)	23 (8.2%)
		Minimum	6.0	0(0.0%)	0(0.0%)	0(0.0%)	0(0.0%)	0(0.0%)	0(0.0%)	0(0.0%)	0(0.0%)
		25th %tile	10.8	7.5 (75.0%)	0(0.0%)	0(0.0%)	0(0.0%)	0(0.0%)	0(0.0%)	0(0.0%)	0(0.0%)
		Median	13.0	11.5 (89.7%)	0(0.0%)	0(0.0%)	0(0.0%)	0(0.0%)	0(0.0%)	0(0.0%)	0(0.0%)
		Average	17.6	14.5 (74.8%)	0.4 (2.7%)	0.4 (3.0%)	0.3 (2.2%)	0.8 (6.5%)	0.8 (3.3%)	0.3 (1.0%)	1.3 (11.1%)
		75th %tile	22.3	17.75 (94.1%)	1 (3.4%)	0(0.0%)	1 (2.0%)	0(0.0%)	0(0.0%)	0(0.0%)	0(0.0%)
		Maximum	51.0	48 (100.0%)	2 (16.7%)	4 (40.0%)	1 (12.5%)	12 (100.0%)	11 (37.9%)	3 (10.3%)	17 (100.0%)

Notes

Data are sorted descending alphabetically, first by country, then by psychedelic substance, and finally by study

Ethnicity reported as “Unknown” for studies in which investigators did not collect self-report data on participant ethnicity. These studies are only excluded from total calculations

Oehen et al. 2013 includes two participants that ultimately dropped out of the study

Moreno et al. 2006 reported 1 participants as ‘Mixed Other,’ categorized above as Mlixed

Indigenous: Native American (USA), First Nation (Canada), Aboriginal (New Zealand)

Mixed: Pardo (Brazil)

Carhart-Harris et al. 2018 only includes participants not included in Carhart-Harris et al. 2018

While 10 of the 17 studies did not initially report ethnicity data, we were able to reach at least one author from each study. In the majority of instances, investigators indicated that although ethnicity data were collected, their country’s reporting guidelines did not require publishing this information. We were therefore able to collect ethnoracial data from all but two Brazilian studies [20, 60] in which data were either not reported or were estimated by study staff (rather than collected through self-report). Collecting ethnicity information in such a manner is well known to be highly inaccurate and problematic methodically [58, 59, 61–63]. Therefore, ethnoracial information for these studies was labeled as unknown and data from these studies were excluded from total calculations in any descriptive statistics.

The final compilation included a total of 282 participants from 16 studies conducted from 2008 to 2017. All of the studies included subjects over the age of 18. Of these, 82.3% of the participants were non-Hispanic White, 2.5% were African-American, 2.1% were of Latino origin, 1.8% were of Asian origin, 4.6% were of indigenous origin, 4.6% were of mixed race, and 1.8% identified their race as “other” (Table 1). Overall there was substantial variability in minority representation, with 13 studies (72.2%) reporting that non-Hispanic White participants accounted for 75–100% of all subjects. Approximately 52.5% of participants were female. Of the seven studies from the United States, all but one [64] were composed of majority White participants. This pattern differed only slightly for studies outside the United States, with two of the eight studies reporting fewer than 75% of participants of non-Hispanic White origin.

In order to better characterize whether such minority inclusion was reflective of minority representation in each respective country, we compared the percent White and people of color in psychedelic studies compared to country-level demographics (Table 2). Data for the two Brazilian studies from which data were not available were excluded in the country-level analysis. Furthermore, attempts to obtain ethnoracial data for Switzerland were unsuccessful as the Statistics Department of that country confirmed that census data only includes information on National origin (i.e. Swiss, Italian, French, German, etc.) and does not include question regarding race or ethnicity. We computed a weighted average across all countries that adjusted for the number of studies included from each country. Overall, while on average, 81.6% of psychedelic study participants were of non-Hispanic White origin, the countries of origin were, on average, 65.4% non-Hispanic White on average. Across all 16 studies, there were 18.4% participants of color on average, compared to country-level representation of 38.2% on average (Table 2).

**Table 2:** Ethnoracial psychedelic studies’ makeup compared to country-level demographics

Country	# Studies	Study Year (s)^2^	Psychedelic Study Enrollment^3^		% Non-White Per Country^5^		Country-Level Demographics^6^	
			% White	% POC^4^	Avg	% White	% POC^4^	Year^7^
Brazil	1	2017	58.6%	41.4%	24.0%	49.0%	53.5%	2010
Canada	1	2013	0.0%	100.0%	24.0%	76.7%	27.7%	2011
Mexico	1	2017	90.0%	10.0%	6.0%	10.0%	90.0%	2010
New Zealand	1	2016	100.0%	0.0%	0.0%	70.0%	34.9%	2013
Spain	1	2008	100.0%	0.0%	0.0%	89.0%	7.9%	2011
Switzerland	2	2013, 2014	92.0%	8.0%	4.0%	N/A	N/A	–
UK	2	2016, 2018	75.0%	25.0%	10.0%	86.0%	14.0%	2011
USA	7	2006–2016	89.0%	11.0%	32.0%	76.9%	39.6%	2016
Total/Weighted Avg/Avg^1^	16	–	81.6%	18.4%	12.5%	65.4%	38.2%	–

Notes

1. Study enrollment averages are weighted by the number of studies by country. Country-level averages are not weighted

2. Reflect range of year (s) of study publications for each country

3. Reflects the total **%** of study participants (white or participants of color) across all studies from each country

4. People of Color

5. Reflects number of participants of color from each country as a percentage of total participants of color across all countries

6. Country-level demographics were compiled for each country separately from census data as reported by the statistics division (census data) of each country. Data are not available for Switzerland, as the country only reports data on nationality and not ethnicity.

7. For each country, reflects census data for percentage of white citizen and people of color. For Mexico, White reflects those solely of European descent; Mestizos (mixed ancestry) are included in the POC column

8. Reflects the publication year of each census data source; Brazil 2010 Population Census (Instituto Brasileiro de Geograpfia e Estatistica) 2011 National Household Survey (Statistics Canada), 2015 Mexico Census (Instituto Nacional de Estadistica y Geografia), New Zealand Census of Population and Dwellings (Stats New Zealand), 2011 Population and Housing Census (Instituto Nacionalde Estadistica), United Kingdom 2011 Census (Office of National Statistics), Bureau), 2016 Census (United States Census).

### Recruitment

All but three studies [21, 65, 66] utilized more than one source of recruitment. The majority of studies (*n* = 12; 66.7%) recruited participants through outpatient physician referrals (Table 3). The second most common approach to recruitment included flyers and local advertisements (*n* = 8; 44.4%) followed by Internet advertisements (*n* = 5; 27.8%) and hospital referrals (n = 5; 27.8%). Only three studies (16.7%) used media advertising and one study [65] recruited from membership organizations.

**Table 3:** Recruitment Methods and Treatment Sites of Psychedelic Medicine Studies

Study	Psychedelic	Recruitment Methods						Treatment Sites
		Outpatient MD Referral	Hospital Referral	Internet Ad Media Ads		Flyer/ Locals Ads	Membership Associations	Location
Osorio et al., 2015	Ayahuasca	✓				✓		Sao Paolo, Brazil
Palhano-Fontes et al., 2017	Ayahuasca	✓		✓	✓			Natal-RN, Brazil
Sanches et al., 2016	Ayahuasca	✓				✓		Sao Paolo, Brazil
Thomas et al., 2013	Ayahuasca		✓					British Columbia, Canada
Brown &Alper, 2017	Ibogaine	✓						Ensenada, Playas de Tijuana, Mexico
Noller, Frampton & Yazar-Klosinski, 2016	Ibogaine	✓						North Island, New Zealand
Bouso et al., 2008	MDMA						✓	Madrid, Spain
Gasseret al., 2014	LSD	✓	✓		✓	✓		Solothurn, Switzerland
Oehen et al., 2013	MDMA	✓	✓					Biberist, Switzerland
Carhart-Harris et al., 2016	Psilocybin	✓						London, UK
Mithoeferet al., 2010	MDMA	✓		✓				South Carolina, USA
Bogenschutz et al., 2015	Psilocybin				✓	✓		Albequerque, NM, USA
Griffiths et al., 2016	Psilocybin	✓		✓		✓		Baltimore, MD, USA
Grab etal., 2011	Psilocybin	✓	✓	✓		✓		Torrence, CA, USA
Johnson et al., 2014	Psilocybin					✓		Baltimore, MD, USA
Moreno et al., 2006	Psilocybin			✓		✓		Tuscon, AZ, USA
Ross et al., 2016	Psilocybin		✓					New York, NY, USA
Carhart-Harris et al., 2018	Psilocybin	✓						London, UK
*N* = 18	Count (%)	12 (66.7%)	5 (27.8%)	5 (27.8%)	3 (16.7%)	8 (44.4%)	1 (5.6%)	

MD Medical Doctor

Hospital Referral includes presentations at local medical or psychiatric hospitals or health offices

Membership associations include women’s groups, mental health support groups and similar private organizations

Excluding the two studies from Brazil that did not collect ethnicity data, we performed unpaired t-tests to determine if there was a significant difference in recruitment strategies between those studies with minority inclusion higher than 20% (*n* = 4) compared to those with less than 20% (*n* = 12). There was also no significant difference in physician referral (*t* = 0.22, *p* > 0.05), hospital referral (*t* = 0.39, *p* > 0.05), use of advertisements (*t* = 0.15, *p* > 0.05) or membership referral (*t* = 0.59, *p* > 0.05) between psychedelic medicine studies with higher minority inclusion compared to those with lower rates of inclusion. Therefore, it does not appear that recruitment approaches contributed to or can explain differences in the rate of minority inclusion.

### Study methodologies

There was considerable methodological variability across studies. The majority of psychedelic medicine trials (*n* = 14, 77.8%) analyzed data quantitatively, although three studies were observational in nature and one only provided qualitative findings. Over half (*n* = 10, 55.6%) included a randomized design with a control group, although the majority of these studies (*n* = 6) did not administer an active placebo. The use of an appropriate placebo that mimics the physiological experiences of psychedelics without the psychological and neurobiological consequences is an important methodological issue in designing double-blinded clinical trials (citation needed). Nine of the 18 studies (50.0%) were double-blinded, three studies (16.7%) were single-blinded and six (33.3%) were open-label trials. While it is likely that some of this variability may be driven by country-specific differences in research practices, reporting requirements, or the current stage of psychedelic medicine research, overall the results suggest that approaches to psychedelic medicine trials are not consistent and have not reached the universal best practices of human clinical trials.

## Discussion

### Minority inclusion and recruitment

In the present study, upon a comprehensive review of psychedelic-assisted psychotherapy studies conducted from 2000-present, we report a lower rate of representation of people of color compared to non-Hispanic Whites. Of the total participants included in this review (*n* = 282), only 2.5% were African-American, 2.1% were of Latino origin, 1.8% were of Asian origin, 4.6% were of indigenous origin, and 4.6% were of mixed race. These data are low when compared to the proportional amounts needed to represent the population, even when considering country-specific differences. The rates are also low when compared to national rates for minority participation in US biomedical research. In 2012, the NIH reported that African-Americans represented 20.6% of all enrollments in NIH clinical research trials, while Hispanics represented 20.0%, and Asian comprised 3.9% of all participants [67].

Notably, some studies did not collect racial and ethnic information and others simply did not report this data upon publication. It is important to note that differences in reporting are likely due, at least in part, to variability in country-level reporting requirements. Not only were ethnicity data not available for specific studies, although in some instances, such information was not even collected on a national census level, likely reflecting differing conceptualizations on the classification and perhaps importance of race and ethnicity. In the case of Switzerland, data were reported based on either nationality (for current citizens) or by immigration status (for non-citizens), without any consideration of ethnicity. Mexico reported data based on geographic regional diversity, but does not collect race or ethnicity data in its census. It is likely that this variability in how ethnicity is defined and viewed in these countries is an important factor in why certain studies did not disclose such information, and speaks to a broader question regarding ethnic and national identity beyond the scope of this body of research. Yet such differences will likely impact any efforts to increase minority recruitment when designing, conducting, and publishing psychedelic science research.

### Potential explanations

One factor for low minority representation in psychedelic studies is due to the lack of cultural inclusivity fostered by the research community. While the DSM-5 has provided a more extensive discussion of culture than previous editions, cultural factors are limited to the Cultural Formulation Interview (which is listed as an emerging measure) and a glossary of cultural concepts of distress found in the appendix [68]. Current symptom dimensions/presentations and treatment protocols listed in the DSM-5 often do not account for cultural variations that shape the clinical presentation of psychopathology in people of color. For instance, the current diagnostic criteria for PTSD in the DSM-5 does not include race-based trauma, which has produced PTSD symptoms similar to other traumas typically characteristic under criterion 1 for PTSD [69]. Without culturally inclusive diagnostic criteria, minorities often do not qualify for treatments studies because their symptom presentations differ from current diagnostic conceptualizations. The inclusion of race-based trauma in the DSM-5 PTSD diagnostic criteria would be especially valuable for MDMA-assisted psychotherapy studies, which have demonstrated efficacy in the treatment of PTSD but have historically struggled to recruit sufficient minority samples [23, 70]. Without expanding such criteria and opening up studies to minorities, it will be unclear whether the efficacy of MDMA-assisted psychotherapy may extend to the treatment of race-based trauma [24]. In order to generalize to the broader population, the field must demonstrate that these therapies are safe, efficacious, and effective in individuals from differing cultural backgrounds.

Ineffective recruitment methods are likely another leading contributor of low minority participation in research. In the present study, the primary recruitment method was “referrals from outpatient providers,” which was used far more frequently than the other methods. “Outpatient providers” could have included physicians and/or mental health clinicians. If the outpatient providers were primarily mental health clinicians, minorities may have been excluded by default. When many individuals of color seek medical care for mental health problems, they often may describe or express their symptoms differentially compared to White patients. For instance, Asian Americans often report somatic symptoms rather than psychological distress [71] and many individuals of Hispanic/Latino origin experience distinct variations in symptoms [68] that may be missed by certain health care providers [72]. Recruitment strategies need to consider cultural variations in symptomatology. Minorities are also less likely to seek psychological care due to the associated stigma or emphasis on other routes of healing (church, family, etc). Furthermore, minorities may only be able to afford physicians, leaving no funds for psychological care. Consequently, recruitment methods should target physician referrals and psychological referrals roughly equally. Seeking referrals from outpatient providers who accept patients with Medicaid and other forms of affordable health care would also increase the probability of recruiting minorities [73]. A variety of other recruitment methods in addition to referrals should also be utilized. Multiple culturally specific advertising strategies and forming genuine, lasting relationships with members of minority communities have been found to be critical in effective recruitment of minorities [73].

Another important factor is the lack of ethnic diversity among psychedelic researchers. Historically, psychedelic research has been predominated by White men and has had few people of color, or women, in positions of leadership (e.g., [3]). In general, pairing minority participants with clinicians of the same ethnoracial background has been shown to improve the treatment process [74, 75]. This may be especially true when a client discloses race-based trauma. A clinician of a race other than the client’s may dismiss the impact race-based trauma has had on the client’s mental health [76]. Such a dismissal could occur if a clinician fails to ask about race-based trauma, only addresses discrete racial trauma, such as a hate crime, rather than cumulative traumatic instances, or unknowingly commits microaggressions against the client [24]. This is not only an issue of representation within the field but of acknowledging the contributions of indigenous people and people of color in advancing the field, and directly involving these communities in the design, recruitment, and implementation of clinical trials. Only by including researchers with specific expertise in areas of cultural diversity and recruitment of people of color will the field be able to increase minority participation and begin to better understand the specific barriers that may be limiting minority participation.

Low minority participation in psychedelic-assisted therapy studies could also indicate minority aversion and resistance. Injustices committed against people of color in the name of medical research are not easily forgotten or forgiven; the Tuskegee Syphilis Study, one of the many harrowing displays of unforgivable abuse and exploitation of minority research subjects at the hands of predominantly White researchers and government, alone accounts for an immense amount of fear minorities still feel about participating in research today [41]. Minorities fears related to being administering drugs may be even more intense when the treatment involves controlled substances, given historic and current inequities in the criminal justice system for drug-related offenses. In addition to fear of criminal actions, minorities may be wary of the physical and mental consequences associated with psychedelic use, including impaired control and cognition increased susceptibility [77–79] and “loss of boundaries between the subject and the objective world” [80]. While any participant may experience concerns about how their bodies will react to a psychedelic substance, the added mistrust of researchers from people of color likely exacerbates reluctance to participate in such studies.

### Limitations

There are several limitations to the present study. Firstly, although efforts were made to summarize minority inclusion rates across studies conducted in different countries, such a direct comparison is difficult given that the minority status of ethnoracial groups differs significantly across nations. Furthermore, factors contributing to minority inclusion, such as mental illness stigma, access to treatment, recruiting practices, and attitudes towards psychedelic use differ substantially between countries and across cultures. In an effort to address this potential confound, country-level census data were included to understand minority participation relative to ethnicity representation in each country. However, several countries do not collect or report ethnicity data in their census (only including nationality or immigration status). In summarizing ethnicity data across differing nations, is not intended to imply equivalency in the experiences and inequities associated with minority status. It is likely that minority recruitment is strongly influenced by these important differences.

The large variability in research design across studies is another limitation to the current review. Over half of the studies did not include a control group (which would have required large samples sizes). Yet differences in methodology appeared to be largely unrelated to minority recruitment; of the three observational studies included in the review, one included 100% people of color but the other two had over 90% participants of color. The variability in methodology likely reflects the current state of psychedelic medicine research as an emerging field and international differences in research practices. Importantly, current ongoing clinical trials in the United States are increasingly conducted with double blind, placebo-controlled randomized controlled designs. Differences in study design could limit the generalizability of study findings on treatment efficacy in minorities and may reduce the success of adopting recommendations for improving minority recruitment.

The present review was also limited by a small sample size. Perhaps more so than other clinical trails, psychedelic research faces significant challenges, ranging from obtaining funding (which in the US has been limited to private sources), obtaining approval to administer scheduled substances, the stigma of mental illness, and stereotypes surrounding psychedelic use. This review was limited to psychedelic-assisted psychotherapy studies and therefore excluded those studies that investigated other aspects of psychedelic medicine (such as psychopharmacology). Many psychedelic studies, including those that are actively addressing concerns related to minority inclusion, are currently ongoing and therefore were not reviewed but raise the possibility that participation rates may become more diversified in the future.

Lastly, the present study was limited to psychedelics that exerted their effects primarily as 5-HT agonist, a definition that resulted in excluding compounds that are being investigated for therapeutic use in psychological disorders and that others [10] have argued should be considered psychedelic in nature. This includes the NMDA-receptor antagonist ketamine, for which there is a growing body of evidence for its efficacy in the treatment of depression [81–84] as well as cannabidol (CBD), a cannabinoid of the cannabis plant with potential uses in the treatment of both medical and psychiatric conditions [11, 12]. There continues to be much debate even within the field of psychedelic medicine regarding the most accurate definition of compounds in this category [10] and therefore the present study aimed to include compounds that were similar form a neurobiological perspective but that were also commonly used in conjunction with psychotherapy. Future studies should investigate the rates of minority inclusion in clinical trials of ketamine and cannabis for the treatment of psychological disorders.

### Current efforts to address inequities

Although the current state of completed research has clearly not addressed the importance of minority inclusion in psychedelic medicine, it would be remiss not to acknowledge current ongoing efforts underway to address these disparities. One of the largest funders and clinical trial organizations for psychedelic studies, the Multidisciplinary Association of Psychedelic Studies (MAPS), has acknowledged the importance of race-based trauma in conceptualization of PTSD and is working with study staff to increase the recruitment of minorities in the Phase III clinical trial of MDMA-assisted psychotherapy for PTSD [24]. MAPS has even added an additional site to the trial that will be specifically focused on recruiting subjects who have experienced race-based trauma. International conferences on psychedelics have also made an increased effort to include panel discussion and guest lectures from indigenous healers and people of color who are not only acknowledging the current state of inequities but are dedicating to helping the field address these issues (e.g., [5]). While these efforts are a promising start, a great deal of additional effort will be needed to translate these steps into increased minority participation and involvement in psychedelic research.

## Conclusions

Currently, the dominant, pervasive image of the psychedelic community is White affluence [85]. This is not only due to the prohibitive costs and lack of access to psychedelic substances but also the glorification of 1960s/1970s White hippie drug use as a “counterculture” rather than an illicit act. The White washing of psychedelic drug use has unfortunately spread to medical research, as we find extremely low rates of participation by people of color in psychedelic-assisted psychotherapy studies and a lack of generalizability of these studies to critical clinical issues for people of color. While several efforts are currently underway to address these concerns, it will take the acknowledgement and efforts of both those without and those with privilege and power to change the field.

Greater inclusion in psychedelic research will require numerous changes to how research is currently conducted. When conducting study visits, researchers should be encouraged to create a culturally inclusive environment, especially in rooms for overnight stays, often encourages minority participants to feel more comfortable. Some researchers suggest diverse artwork and ethnically themed magazines [86] inclusive music should also be considered, especially given the fact that music is often played for more than 6 h straight during overnight stays for MDMA-assisted therapy. Session scripts should use culturally inclusive language in and avoid any jargon that is biased toward White “hippie” culture. Creating equally comprehensible and resonant scripts for participants of other cultures would allow them to feel more comfortable and trusting of the environment. An ethnoracially-matched clinician participating during this process would is also advised for the many reasons stated above.

The economic burden of participating in psychedelics studies may be higher than compared to other clinical treatment studies. The time commitment is often longer and therefore participants may need to take more time off of work or cover additional days of childcare compared to other studies. Psychedelic studies may include several meetings with a therapist in the initial stages, overnight visits, an integration session, and a follow-up session. This effect may be exacerbated for marginalized groups, who have a lower average income than their White counterparts [87]. Compensation should be commensurate with the time, effort, and burden placed on study subjects.

The stigma of drug use itself must be directly addressed given the long history of discriminatory drug enforcement practices in the United States. Whites have the privilege of publicizing psychedelic use with lesser consequences than minorities and therefore some participants may feel excluded from these experiences. Even if the psychedelics are administered in a legal, health-oriented setting, minorities may still feel that they are playing out the stereotype that people of color are drug users and engage in illegal activity. Further stigmatization may arise from other community members as minority participants may be criticized for engaging in a “White” treatment.

While the present study provides strong empirical evidence for the lack of minority inclusion in modern psychedelic medicine, the factors that have contributed to this issue are complex. It will be impossible to fully characterize these factors until more minorities are included in the movement. In many regards, the psychedelic medicine movement both exemplifies the existing inequities and barriers to mental healthcare treatment inherit in modern psychiatry, while also presenting an enormous opportunity to acknowledge the efficacy and powerful contributions of indigenous medicine and rectify the injustices of the past (George et al., in press). However, it will only be successful in doing so to the extent that those with power acknowledge the importance of this issue and consciously make an effort to address the concerns presented herein.

## Abbreviations

DMT: N,N-Dimethyltryptamine
DSM-5: Diagnostic and statistic manual of mental disorders fifth edition
LSD: Lysergic acid diethylamide
MAPS: Multidisciplinary Association for Psychedelic Studies
MDMA: 3,4-Methylenedioxymethamphetamine
NIH: National institute of health
OCD: Obsessive compulsive disorder
PTSD: Post-traumatic stress disorder
US/USA: United States of America
